# Multiple talker processing in autistic adult listeners

**DOI:** 10.1038/s41598-024-62429-w

**Published:** 2024-06-26

**Authors:** Samra Alispahic, Elizabeth Pellicano, Anne Cutler, Mark Antoniou

**Affiliations:** 1 The MARCS Institute for Brain, Behaviour and Development, Western Sydney University, Sydney, NSW Australia; 2 Department of Educational Studies, Macquarie University, Sydney, Australia; 3 Department of Clinical, Educational and Health Psychology, University College London, London, UK; 4 Max Planck Institute for Psycholinguistics, Nijmegen, The Netherlands; 5 ARC Centre of Excellence for the Dynamics of Language, Clayton, Australia

**Keywords:** Psychology, Human behaviour

## Abstract

Accommodating talker variability is a complex and multi-layered cognitive process. It involves shifting attention to the vocal characteristics of the talker as well as the linguistic content of their speech. Due to an interdependence between voice and phonological processing, multi-talker environments typically incur additional processing costs compared to single-talker environments. A failure or inability to efficiently distribute attention over multiple acoustic cues in the speech signal may have detrimental language learning consequences. Yet, no studies have examined effects of multi-talker processing in populations with atypical perceptual, social and language processing for communication, including autistic people. Employing a classic word-monitoring task, we investigated effects of talker variability in Australian English autistic (*n* = 24) and non-autistic (*n* = 28) adults. Listeners responded to target words (e.g., *apple*, *duck*, *corn*) in randomised sequences of words. Half of the sequences were spoken by a single talker and the other half by multiple talkers. Results revealed that autistic participants’ sensitivity scores to accurately-spotted target words did not differ to those of non-autistic participants, regardless of whether they were spoken by a single or multiple talkers. As expected, the non-autistic group showed the well-established processing cost associated with talker variability (e.g., slower response times). Remarkably, autistic listeners’ response times did not differ across single- or multi-talker conditions, indicating they did not show perceptual processing costs when accommodating talker variability. The present findings have implications for theories of autistic perception and speech and language processing.

## Introduction

Accommodating the speech of multiple talkers is a complex and multi-layered cognitive task. It involves processing not only indexical characteristics of the talkers (e.g., differences in age, gender, emotion, social stature, dialect etc.^1^), but also the content of their speech. These processes in turn affect and interact with attention and cognitive load demand^2^. As phonetic and voice processing are highly interdependent^3^, challenges attending to either may have detrimental consequences for language learning, particularly for people who have altered phonetic perceptual patterns. For instance, autistic adults are able to perceive speech but exhibit relatively select voice processing atypicalities^4^. How these atypicalities interact with their ability to accommodate multiple talkers remains unclear.

It was once thought that variation between the voices of different talkers was unimportant, and that talker-specific information was discarded during the act of speech perception^5–9^. We now know, however, that talker information is not discarded because it results in a reliable processing cost during speech perception^10^. Many studies have demonstrated that processing the speech of multiple talkers is generally slower (and sometimes less accurate) compared to that of a single talker^11–14^. Such talker variability effects occur irrespective of whether the talkers are novel or familiar to the listener^15^.

While several theoretical perspectives have been proposed to account for multi-talker accommodation^16–18^, the most prominent view has been termed *talker normalisation*^19–21^. According to this view, as the speech signal unfolds, listeners extract information about a talker’s vocal characteristics^1,22^. The listener maps the speech produced by the talker to internal phonetic categories^23^. The processing required to recalibrate the vocal characteristics of multiple talkers is cognitively demanding^20^. Therefore, the well-established processing costs of accommodating talker variability may be due to the increased cognitive demands required by talker normalisation^24^, that is, the constant recalibrating between differing talkers’ speech characteristics^25,26^. To date, researchers have not investigated how resolving multi-talker speech affects task performance in people who may exhibit atypicalities relative to social and speech processing, namely autistic people.

Autism affects the way a person interacts and communicates with others^27^. While theoretical accounts have not been extended to account for speech processing, existing theoretical and empirical work offers three competing possibilities for how autistic listeners might respond to multi-talker listening environments. First, atypical voice processing has been attributed to diminished motivation to process social information in autism^28,29^. Reduced activity in the superior temporal sulcus^30^, a core brain region subserving processing of the human voice^31^, has been interpreted as possible “indifference” to the voices of others^32,33^. Second, two related but distinct accounts propose a ‘detail-focused’ style of information processing, as set within Weak Central Coherence^34^, and/or preference of lower-level processing, as described by Enhanced Perceptual Functioning^35^. Such differences may affect higher-level language processing, resulting in superior perceptual processing of fine-grained voice information^36,37^, but atypical speech sound specialisation or categorisation involving more complex voice stimuli, such as syllables^38–40^.

The third possibility is that processing differences may arise from atypical predictive adaptation as derived within computational accounts (Bayesian decision theory^41–43^), relative to processing of complex or higher-level social stimuli^44–46^. Perceptual adaptation involves continuous (re)calibration of incoming sensory information, which match and/or update intrinsic statistics of the input^47^—and these adaptive mechanisms are believed to differ in autistic people^41,48^. Given that different talkers produce markedly different speech sounds, the listener is required to calibrate the incoming speech to the categories with the highest response probabilities^49^. Such estimation of probabilities during perception are believed to differ in autistic people^50^. While prediction differences have recently been proposed to be the underlying cause of language learning differences observed in autistic people, their influence on spoken language processing in autism has not been systematically investigated^51^.

Multi-talker speech introduces added acoustic–phonetic variability, and the resulting ambiguity affects perceptual efficiency in non-autistic listeners^23,52–55^. However, it remains unclear how efficiently autistic listeners adapt to novel and varying voices. Autistic adult listeners’ neural processing of word-and-sentence level spoken language appears to be similar to non-autistic adults’, in both clear and noisy environments^56,57^. Likewise, processing of social characteristics of voices such as gender or age have been shown to be unaffected in autistic listeners^58^. Yet, voice processing difficulties become apparent during discrimination, learning and recognition of novel compared to familiar talkers^4,59,60^. Voice identity learning difficulties have been linked to neural differences in autistic listeners’ abilities to integrate structural lower-level voice information into a coherent percept^61^. If experience-dependent neural adjustment differs in autism then autistic listeners’ perceptual efficiency may also differ, relative to novel multi-talker speech. There is some evidence to suggest that autistic listeners might not show the same processing costs observed in non-autistic listeners. For example, autistic listeners are faster than non-autistic listeners when processing sung vowels by a novel talker, suggesting processing differences of vocalised sounds^62^.

Accommodating talker variability requires the allocation of additional cognitive resources (e.g., working memory), which in the general population leads to slower performance and lower accuracy in perceptual tasks^63^. Given the limited findings concerning voice processing in autism, it is unclear how autistic people process single- versus multi-talker speech. Here, we addressed this gap by investigating multi-talker adaptation in Australian English autistic and non-autistic adults via a word-monitoring task, in which common words were spoken in two key conditions—by either (1) a single talker or (2) multiple talkers. In so doing, we tested the following three competing hypotheses:

i. **Global Processing Difficulty**: Consistent with the common function of the superior temporal sulcus in social and speech processing, autistic adults should show a general processing difficulty, that is, prolonged response times across *both* single- and mixed-talker conditions^30^ relative to non-autistic listeners;

ii. **Exaggerated Cost Hypothesis**: Due to a tendency to focus on local details, thereby inducing higher levels of perceived novelty to stimuli, autistic adults should show greater processing costs (i.e., prolonged response times) when accommodating talker variability than non-autistic listeners^35^;

iii. **No Cost Hypothesis**: Due to differences in social functioning and diminished adaptation involving prior higher-level social stimuli, autistic adults’ responses should fall within the range of non-autistic participants, but they should not show the usual processing cost associated with talker variability^41^.

## Methods

### Participants

Australian English-born monolingual autistic (*n* = 24; 5 females) and non-autistic adults (*n* = 28; 22 females) completed this experiment. This study was conducted with the approval of Western Sydney University’s Human Research Ethics Committee. All participants provided written and verbal informed consent prior to participation, reported no hearing difficulties, and were paid a small fee (AUD$60) for their time. Data from one additional participant (AUT group) were excluded due to an experimental script-error. The autistic and non-autistic groups were not matched for gender, *t*(50) = 4.537, *p* < 0.001, Cohen’s *d* = 1.262. Autistic participants (*M age* = 22.0, *SD* = 8.1, range = 18–48 years) were significantly younger than the non-autistic participants (*M age* = 30.3, *SD* = 6.8, range = 19–48 years), *t*(51) = 3.987, *p* < 0.001, Cohen’s *d* = 1.109. All participants were born and raised in Australia, and were monolingual speakers of English. Participants’ demographic information is presented in Table 1.

**Table 1:** Demographic information and measures of participants in the autistic and non-autistic groups.

Group	Autistic	Non-autistic
*M* (SD; range) N (%) or frequency		
N	24	28
Female/male	5/19 (21/79%)	22/6 (79/21%)
Native language	Australian English	
Formal diagnosis of autism	24	0
Age of diagnosis (in years)		
0–11	19	0
12–18	2	0
18 +	3	0
Other self-reported diagnoses		
Anxiety	15 (63%)	11 (39%)
Depression	8 (33%)	9 (32%)
Attention-deficit/hyperactivity disorder (ADHD)	8 (33%)	0
Dyslexia	2 (8%)	1 (4%)
Obsessive–compulsive disorder (OCD)	3 (13%)	0
Highest level of education		
School certificate	4 (17%)	0
Higher school certificate	8 (33%)	5 (18%)
Technical and further education	10 (42%)	6 (21%)
(TAFE) certificate		
Undergraduate (university)	2 (8%)	9 (32%)
Postgraduate (university)	0	8 (29%)
Currently employed		
Yes	8 (33%)	24 (86%)
No	16 (67%)	4 (14%)
Previously employed		
Yes	13 (54%)	26 (93%)
No	11 (46%)	2 (7%)
Average of hours (per week) spent interacting with people outside of immediate circle of family/friends	3.17 (SD 3.088; range = 1–15)	18.25 (SD 3.884; range = 3–20)

Table 2 includes participants’ age in years, and measures of intellectual functioning (Wechsler Abbreviated Scale of Intelligence—Second edition)^64^, and autistic traits (Social Responsiveness Scale—Second Edition; SRS-2)^65^. No between-group differences were observed in intellectual functioning across full-scale IQ, perceptual reasoning (PCI), or verbal comprehension (VCI) (all *p* > 0.242). However, as expected, SRS-2 scores were significantly higher in autistic compared to non-autistic participants, consistent with clinically-significant autistic features, *t*(51) = 8.425, *p* < 0.001, Cohen’s *d* = 2.344.

**Table 2:** Comparison of the autistic (AUT) and non-autistic (non-AUT) groups in terms of their age, SRS-2, FSIQ-4, VCI and PRI scores, and interaction hours per week.

	Group	N	Min	Max	M	SD	SE	T	p	d
Age in years	AUT	24	18	58	22.04	8.084	1.65	3.987	< 0.001*	1.109
	Non-AUT	28	19	48	30.25	6.764	1.278			
SRS-2 score	AUT	24	50	79	66.88	7.903	1.613	− 8.425	< 0.001*	2.344
	Non-AUT	28	40	63	48.82	7.528	1.423			
FSIQ-4	AUT	24	94	146	117.67	15.07	3.076	− 0.557	0.580	− 0.155
	Non-AUT	28	100	131	115.75	9.474	1.79			
VCI	AUT	24	100	160	121.25	15.771	1.861	− 0.539	0.242	− 0.329
	Non-AUT	28	102	139	117	9.847	1.861			
PRI	AUT	24	86	154	112.54	17.151	2.056	− 0.593	0.556	− 0.165
	Non-AUT	28	94	133	110.21	10.881	2.056			
Interaction hours p/week	AUT	24	1	15	3.17	3.088	0.630	− 18.35	< 0.001*	4.261
	Non-AUT	28	3	20	18.25	3.884	0.734			

Effect size reported in Cohen’s d with significant group differences (p < .05) marked with an asterisk (*).

Age in years; social responsiveness scale (SRS‐2) score; full scale IQ (FSIQ-4); verbal comprehension (VCI); perceptual reasoning (PCI); and hours spent interacting with people outside of their immediate circle of family and friends per week (Interaction hours p/week) were included as variables in the group statistics.

### Stimuli and procedure

#### Stimulus materials

Stimuli in the word-monitoring task were 24 concrete nouns, that have been used in prior research^25,66^. There were eight target words (*apple*, *bear*, *bin*, *cat*, *corn*, *duck*, *fish*, and *horse*), and 16 filler words (*chalk*, *clock*, *lettuce*, *mushrooms*, *notebook*, *onion*, *orange*, *parrot*, *pencil*, *rabbit*, *scissors*, *squirrel*, *stapler*, *strawberries*, *tape*, and *watermelon*). The words were recorded (16-bit, 44.1 kHz) from eight English monolingual talkers (4 males, 4 females). For each word, stimulus tokens were selected that had comparable durations across talkers. Tokens were root-mean-square normalised to an output level of 72 dB SPL.

#### Procedure

Participants were told they would be completing a word-monitoring task. At the beginning of each trial sequence, participants were presented with a target word displayed on screen in a large font. They were instructed to memorise the target word and, when ready, press the spacebar to begin. The target word would then disappear leaving a blank screen and a sequence of words would be heard through headphones. Participants were instructed to press the spacebar whenever the target word was heard. Each sequence lasted approximately 30 s, with an inter-stimulus interval of 250 ms between words. A new sequence would be indicated to the listener whereby a blank screen would flash and a new target-word would be displayed. Participants were asked to respond as quickly and accurately as possible and, if they became aware they made an error, to simply continue until the end of the experiment. This procedure closely modelled previous research^20,25,66^.

Each sequence comprised four presentations of the target word (+ 23-filler words = 27 words per sequence). In total, sixteen sequences were presented in pseudo-randomised order: half produced by a single talker; the other by multiple talkers. Multi-talker sequences included two presentations of the target word produced by one male and one female talker, while filler words were produced by the other six talkers. Words within each sequence were randomised, but had the following constraints: target words were always separated by at least one filler; target words could not occur in the first or last position. Single- and multi-talker sequences alternated during the experiment, with single-talker sequences always being presented first.

Participants’ first sequence responses were omitted from analyses to ensure that the data reflected performance only when they had become familiar with the experimental procedure (Note that we also conducted all statistical analyses with all data points included and the pattern of results was the same as those reported.). Sensitivity scores (*d’*) were computed using the formula *z*(hit rate) – *z*(false alarm rate). Participants’ response times for correctly identified target words were also calculated. Correct hits were defined as registered keypresses following the presentation of a target word, while false alarms were keypresses following a filler word. Responses were accepted up to 800 ms after the offset of the target word. Applying this criterion resulted in two responses being excluded (out of 3328 = 0.06% excluded).

### Ethics statement

Ethics approval was obtained by the University’s Human Research Ethics Committee (H3315).

## Results

**Figure 1:**
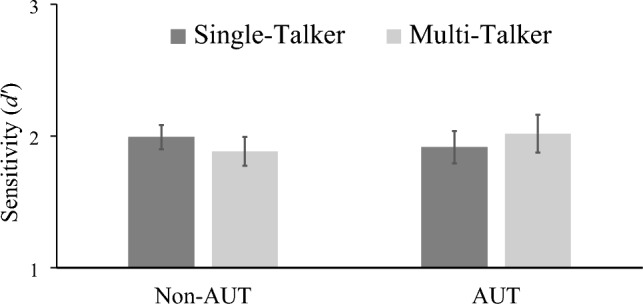
Non-autistic and autistic participants' sensitivity scores (d') for correctly spotted target words. Error bars represent standard error of the mean (SEM).

Autistic and non-autistic participants’ word-monitoring sensitivity scores (*d'*) are illustrated in Fig. 1.

First, a 2 × 2 analysis of variance (ANOVA) was conducted on the word-monitoring sensitivity scores (*d'*), with the between-subjects factor of group (autistic vs. non-autistic) and the within-subjects factor of talker variability (single- vs. multi-talker). The analysis revealed no significant main effects of talker variability, *F*(1, 50) = 0.001, *p* = 0.975, ${\upeta }_{p}^{2}$ =  < 0.001, or group, *F*(1, 50) = 0.044, *p* = 0.834, ${\upeta }_{p}^{2}$ = 0.001. There was also no significant interaction between talker variability and group, *F*(1, 50) = 1.37, *p* = 0.247, ${\upeta }_{p}^{2}$ = 0.027. Sensitivity (*d’*) scores for correctly identified words did not differ between groups.

**Figure 2:**
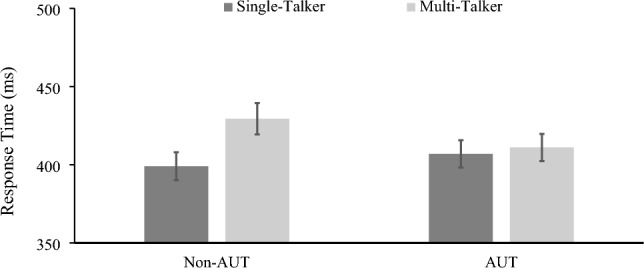
Autistic and non-autistic participant's response times to words produced in single and multi-talker conditions. Error bars represent standard error of the mean (SEM).

A second, 2 × 2 ANOVA was conducted on participants’ response times for correctly identified words. There was a significant main effect of talker variability, *F*(1, 50) = 7.96, *p* = 0.007, ${\upeta }_{p}^{2}$ = 0.137, reflecting faster response times in the single-talker compared to the multi-talker condition. There was no significant main effect of group, *F*(1, 50) = 2.06, *p* = 0.652, ${\upeta }_{p}^{2}$ = 0.004, that is, autistic and non-autistic participants did not differ in their overall response times, suggesting that the autistic listeners’ responses fell within the normal range. There was, however, a significant interaction between talker variability and group, *F*(1, 50) = 4.59, *p* = 0.037, ${\upeta }_{p}^{2}$ = 0.084, suggesting that autistic and non-autistic listeners showed different patterns of word spotting speed across the single- versus multi-talker conditions (see Fig. 2).

To examine this interaction, we conducted Bonferroni-adjusted *t*-tests with α set to 0.025. These confirmed that the non-autistic group showed the well-established cost associated with multi-talker speech processing, *t*(27) = 3.5, *p* = 0.002, Cohen’s *d* = 0.664. That is, non-autistic listeners responded faster for sequences spoken by a single talker (399 ms, *SD* = 47.4) than multiple talkers (429 ms, *SD* = 52.9). In contrast, autistic listeners *did not* show similar costs due to talker variability: for the autistic participants, there was no significant difference between response times for target words produced by a single (*M* = 407 ms, *SD* = 40.6) or by multiple talkers (*M* = 411 ms, *SD* = 42.7), *t*(23) = 0.487, *p* = 0.631, Cohen’s *d* = 0.099. To further explore whether the two groups were affected differently by talker variability, we conducted an additional *t*-test on the difference between their response times on the single- and multi-talker conditions. This revealed a significant difference, *t*(44.93) = − 2.148, *p* = 0.019, *d* = − 0.577, confirming that the non-autistic group were reliably affected by talker variability (*M* diff = 30.3 ms, *SD* = 45.7) whereas the autistic group were not (*M* diff = 5.8 ms, *SD* = 42.1).

## Discussion

The present study investigated the processing of multi-talker speech by autistic and non-autistic Australian-English adult listeners, testing three competing hypotheses: (i) Global Processing Difficulty; (ii) Exaggerated Cost Hypothesis; and (iii) No Cost Hypothesis. Our findings revealed that non-autistic adults showed the well-established processing costs associated with accommodating the speech of multiple talkers, namely prolonged response times relative to single-talker processing. Strikingly, the autistic group did not show this well-established pattern: for autistic listeners, response times did not differ when they processed the speech of single versus multiple talkers, despite showing accuracy levels that were indistinguishable from their non-autistic counterparts. These findings suggest an autistic advantage in processing time during multi-talker scenarios, at least as compared to non-autistic listeners.

Since autistic listeners’ response times fell within the typical range of the non-autistic listeners, we found no support for the Global Processing Difficulty hypothesis. We also failed to find support for the Exaggerated Cost Hypothesis. Earlier accounts of speech perception in autistic people stated a preference for lower-level processing^35^. From this view, if our autistic listeners were sensitive to the perceptual novelty of a talker-change, this would have prolonged response times when processing multi-talker speech. However, our autistic listeners’ response times did not differ between single- and multi-talker conditions. Rather, the current results lend support to the No Cost Hypothesis, that is, differences in the use of prior higher-level social stimuli appear to confer no talker variability cost for autistic adults.

The current findings demonstrate differences between autistic and non-autistic adult listeners in the use of available speech information. Processing of complex higher-level speech was comparable in terms of sensitivity scores between listener groups; however, during spoken word recognition, talker variability led to a delay in response times in non-autistic listeners, but did not incur a similar processing cost in autistic listeners. The present findings are consistent with accounts of atypical incorporation of higher-level and lower-level processing, as set out within the Bayesian framework^41^. They are also in line with previous reports of attenuated perceptual mechanisms involving prior social information^67^, and atypical predictive adaptation^68^. Current findings further support earlier accounts of learning built through a lifetime of experience in the autistic population and atypical predictive coding integration of relevant information in spoken word processing^69^.

Existing studies investigating speech processing by autistic listeners have not investigated cognitive load and response times relative to speech produced by multiple talkers^70–74^. Cognitive demands during spoken word processing are higher when speech is produced by multiple talkers^75^. A difference in rapid calibration involving higher cognitive load demands may therefore by proxy be advantageous for some autistic listeners. Therefore, and at *no cost* of accuracy, autistic individuals may be less distracted by talker variability. One explanation is that this may be evidence for an increased cognitive load capacity (i.e., working memory), as demonstrated in earlier visual inhibition tasks^76^ and increased auditory perceptual capacity in detecting non-speech sounds compared to non-autistic listeners^77^.

Atypical hierarchical encoding of speech may lead to differences in the speed and robustness of the early stage of auditory change detection in autistic people^78,79^. When perceiving the speech of different talkers, listeners are required to accommodate talker variability in order to understand the linguistic utterance^55^. This adds an additional processing layer that generally leads to prolonged response times, particularly when attending to more complex stimuli (e.g., words as compared to individual speech sounds)^20^. Altered adaptation involving processes of higher-level knowledge (i.e., words), and rapid updating of lower-level linguistic categories, may affect how a new talker is detected and normalised during active listening^80^. The absence of processing costs in the autistic group may therefore indicate that talker change detection differs in autistic people. Differences in talker change detection, may, in turn, affect learning outcomes relative to talker-specific speech characteristics, or when resolving ambiguity within phonetic interpretations. These results are in line with reduced adaptation^46^, slower categorical updating^81^, and atypical phonetic encoding and language learning^82^ previously reported in autistic people.

Successful encoding of talker-related speech characteristics has been shown to have a positive impact on recognition, linguistic processing and long-term memory^83^. The perception of a novel talker’s voice interacts with the acoustic–phonetic analysis of an utterance, and talker-specific mapping is improved the more speech is heard by a listener. Talker familiarity, in turn, facilitates perceptual accuracy in the recognition of spoken words and sentences in both native^17^ and non-native listening^84^. Therefore, the lack of talker variability effects in our autistic listeners may have emerged because they interact with fewer novel interlocutors (see Table 1) than non-autistic listeners. Processing of speech in the auditory system is facilitated by degree of social interaction^85^, and increased input variability relative to social network size improves perception of speech in noise^86^. Likewise, for efficient perceptual adaptation, a regular supply of novel conversational partners is required^87,88^.

Earlier research on speech processing has questioned the impact that social factors may impose on phonological and language development in autistic children^89,90^. Effects of smaller exposure to different talkers within a social environment have been alluded to^45,90^, but not explicitly investigated. Autistic listeners respond faster when attending to sung vowels compared to non-vocal sounds produced by a novel talker, and compared to non-autistic listeners^62^. This does not imply that the ability to learn and recognise voices is absent in autistic people; in fact, in the same study, autistic listeners *outperformed* non-autistic listeners on voice recognition of newly encountered talkers although Lin and colleagues^62^ did not investigate how many people their autistic listeners regularly interacted with. Accounts of altered language development in autism have previously been attributed to social withdrawal^91^. We know, however, that autistic people are interested in social connections with others^92–94^. Thus, investigating environmental contributions to speech perception in autism warrants further investigation.

## Limitations

Due to data collection occurring during stringent COVID-19 lock-downs, the participant groups reported here were not matched in age or gender. Yet, neither gender nor age correlated with sensitivity or response times for either single- or multi-talker conditions (Supplementary Table A), suggesting limited influence of these background variables^16^. Further, due to not being able to access participants in-person for further testing we were also unable to test (1) whether processing patterns of multiple talkers extend to familiar (or trained) voices in autistic participants; and (2) whether a different group of autistic participants that has more in-person experience with a variety of new talkers would show the same perceptual patterns reported here. Nonetheless, the current results offer exciting possibilities for future research.

## Conclusions

In sum, accommodating talker variability has been shown to exert processing costs in non-autistic listeners. However, prior work has highlighted that autistic listeners process voice and speech sounds differently to non-autistics, and this may relate to differences in flexibility during social interactions. Remarkably, our autistic listeners’ performance did not differ across single- or multi-talker conditions, indicating they did not show perceptual processing costs when accommodating the speech of multiple talkers. Future research should extend this work with autistic children and adolescents, and also examine whether the effects of speech processing are relative to the amount of experience with the number of novel interlocutors, familiarity effects as well as the comparison of in-person talker processing with interactions involving other media (e.g., assistive technologies, video, television, artificial intelligence).

### Supplementary Information

Supplementary Table 1.

## Data Availability

The data that support the findings of this study are available in Western Sydney University Research Data open access repository via 10.26183/cpk0-4b92. Alternatively, data supporting the findings of this study are also available from the corresponding author S.A. on request.
