# Supplementary material for: Multiple talker processing in autistic adult listeners
Source: Sci Rep. 2024 Jun 26;14:14698. doi: 10.1038/s41598-024-62429-w (PMC11208580; doi:10.1038/s41598-024-62429-w)
Supplement: Supplementary file 1 — Supplementary Table 1. [file 41598_2024_62429_MOESM1_ESM.docx]

**Supplementary Material**

Ref: Submission ID 444f560d-5aa1-478d-8b55-bcb1a4107dfc

**Table A.** Correlational analyses of participants' gender and age with sensitivity and response times across test conditions.

|  | DPBlk | DPMix | RTBlk | RTMix |
| --- | --- | --- | --- | --- |
| Gender | r(52)=0.062,p=0.664 | r(52)=0.023,p=0.873 | r(52)=0.022, p=0.877 | r(52)=0.031,p=0.828 |
| Age | r(52)=0.149,p=0.291 | r(52)=0.112,p=0.428 | r(52)=-0.246, p=0.078 | r(52)=0.058,p=0.682 |
